# Terahertz optoacoustic detection of aqueous salt solutions

**DOI:** 10.1016/j.isci.2022.104668

**Published:** 2022-06-26

**Authors:** Liwen Jiang, Ke Zhang, Yixin Yao, Shuai Li, Jiao Li, Zhen Tian, Weili Zhang

**Affiliations:** 1 School of Precision Instruments and Optoelectronics Engineering, Tianjin University, No.92 Weijin Road, Nankai District, Tianjin 300072, China; 2 Center for Terahertz Waves, Tianjin University, No.92 Weijin Road, Nankai District, Tianjin 300072, China; 3 Key Laboratory of Optoelectronics Information and Technology (Ministry of Education), No.92 Weijin Road, Nankai District, Tianjin 300072, China; 4 School of Electrical and Computer Engineering, Oklahoma State University, Stillwater, OK 74078, USA

**Keywords:** physics, optics, applied sciences

## Abstract

Terahertz radiation has been used to detect aqueous salt solutions; however, strong absorption of water in terahertz regime limits the application of traditional terahertz techniques. Here, we present a novel method in analyzing aqueous salt solutions via terahertz optoacoustics. Terahertz optoacoustic signals can be manipulated by temperature control, which allows the dampening of water background and providing more information of solute. We demonstrate that dynamic and continuous terahertz optoacoustic detections of salt solutions with different solutes, concentrations, temperatures, and terahertz spectral frequencies shows the significant potential of this method in distinguishing different salt solutions and quantitatively analyzing salt concentrations. Terahertz optoacoustics may be a powerful tool for quantitative and label-free detection of aqueous salt solutions to further study the complicated aqueous solutions in terahertz regime.

## Highlights

• THz radiation and optoacoustics are combined to distinguish various salt solutions
• Quantitative THz-OA detection of salt solutions is realized by temperature control
• THz absorption of low concentration salt solutions is reflected by THz-OA detection

## Graphical abstract

**Figure:**
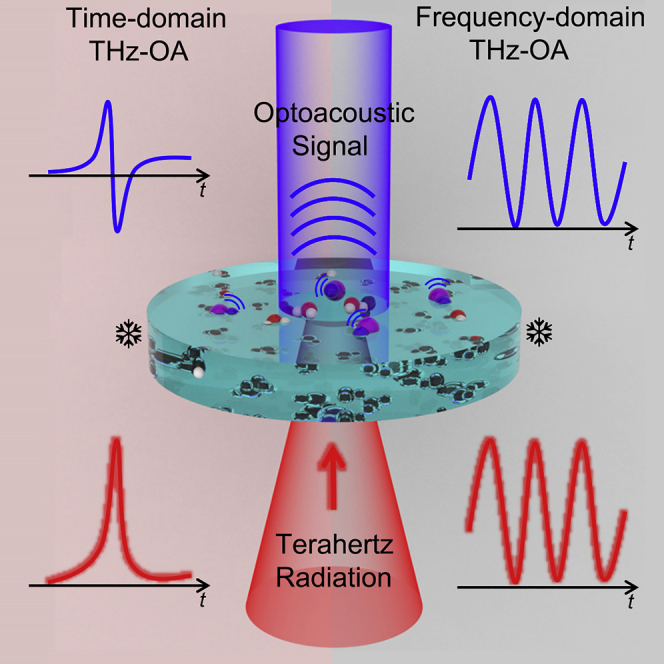

## Introduction

Aqueous salt solution, in which ions dissolved in water produce considerable perturbation of the hydrogen-bond structure of the liquid and bind to water molecules to form hydration shells (Drt, 1982). Salt solutions have specific physical and chemical characteristics, because ion hydration affects the structure and dynamics of water. Since the 19th century, the study on Hofmeister series describes the ability of ions to destabilize (salt out) or stabilize (salt in) egg white and serum proteins in aqueous solutions (Hofmeister, 1888; Zhang and Cremer, 2006). Many researchers believe that the Hofmeister series reflects the long-range structuring of water by specific ions kosmotropes (structure makers) versus chaotropes (structure breakers) (Marcus, 2009). However, there are some studies that suggest that the ions may be treated as simple defects in the water H-bond network, therefore cannot be characterized as either kosmotropes or chaotropes (Schmidt et al., 2009). These controversial opinions show that obtaining a complete understanding of salt solutions still remains challenging due to the physical and chemical complexities involved in it. Complementary techniques have been applied to study the salt solution, such as neutron and X-ray diffraction (Mancinelli et al., 2007; Gaspar et al., 2004), X-ray absorption spectroscopy (Cappa et al., 2006; Klewe and Pedersen, 1974; Reddy et al., 2016), Infrared and Raman spectroscopy (Smith et al., 2007; Bergstroem et al., 1991) and dielectric spectroscopy (Buchner and Hefter, 2009; Buchner et al., 1999; Chen et al., 2003; Lyashchenko and Lileev, 2010). In consideration of that the spectral response of hydrogen-bond network is in terahertz (THz) regime, THz radiation (wavelengths between 0.03 and 0.3 mm) has been used to analyze the characteristics of water and salt solutions (Tonouchi, 2007; Jin et al., 2020; Funkner et al., 2012; Schwaab et al., 2019).

THz spectroscopy is capable of probing collective motions that hydrogen bonds are formed and broken on picosecond time scales, offering a useful tool to study the features of hydration network in aqueous solutions (Schmidt et al., 2009; Mc Intosh et al., 2012; Ueno and Ajito, 2008; Takahashi, 2014). Several studies have utilized the time-domain THz spectroscopy to obtain THz absorption information of salt solutions in liquid state and frozen state, in order to explore the signatures of ion-water systems (Chen et al., 2020). However, the applications of THz technique on aqueous solutions face tough challenges caused by the strong absorption of water in the THz regime. Although THz reflectance spectra or transmissive THz spectroscopy with extremely thin samples have been employed to reduce background water (Brun et al., 2010), the complicated pretreatment of samples limits their dynamic and precise detection of aqueous solutions. In addition, strong absorption of water in aqueous solutions drowns out weaker signals from solutes. Thus, it has been a bottleneck hindering revealing physical and chemical features and quantitative detection of aqueous solutions by THz radiation.

In our recent work, we present a novel method in analyzing aqueous salt solutions via time-domain terahertz optoacoustics (THz-OA), breaking the limitation of complicated pretreatment of aqueous solution samples and dampening the strong optoacoustic signals of water background (Li et al., 2021). Here, we further combine time-domain THz-OA and frequency-domain THz-OA to study the character of aqueous solutions of nine alkali halides and three alkaline earth metal halides, and figure out the relationship between THz-OA signal and the relative physical parameters, such as solute concentration, temperature and THz spectral frequency. Dynamic and continuous THz-OA detections of salt solutions show the significant potential of this method in distinguishing, quantitatively detecting different salt solutions with low concentrations by reflecting their THz absorption.

## Results

**Figure 1:**
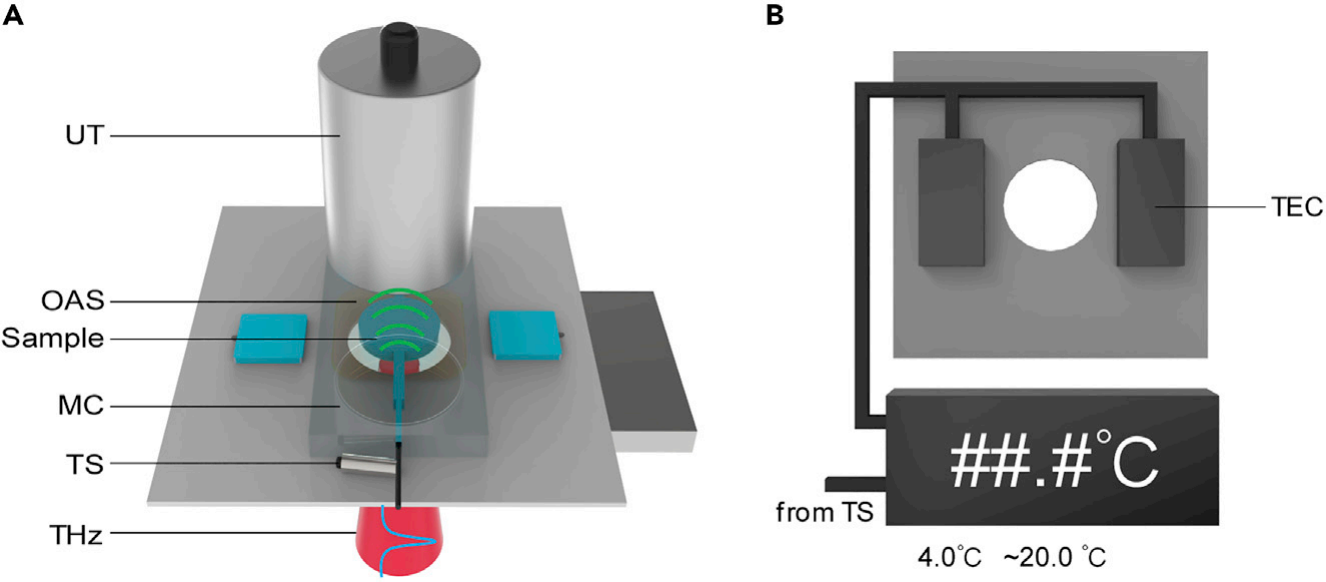
Schematic for time-domain THz-OA measurement (A) Schematic of the setup. UT, ultrasonic transducer; OAS, optoacoustic signal; THz, terahertz radiation; MC, microfluidic chip; TS, temperature sensor; A peristaltic pump drives water into the microfluidic chip at the inlet, and the water exits at the outlet. (B) Schematic of the temperature control module. TEC, thermoelectric cooler.

The time-domain THz-OA system, as shown in Figure 1, presented here incorporates terahertz radiation source, sample holder, temperature control module, piezoelectric ultrasonic transducer (system details see STAR Methods). The THz-OA signals are detected by ultrasonic transducers after the interaction between terahertz radiation and samples. The aqueous salt solutions to be detected are circulating through customized microfluidic chips in order to realize dynamic and continuous detection at the selected temperature.

**Figure 2:**
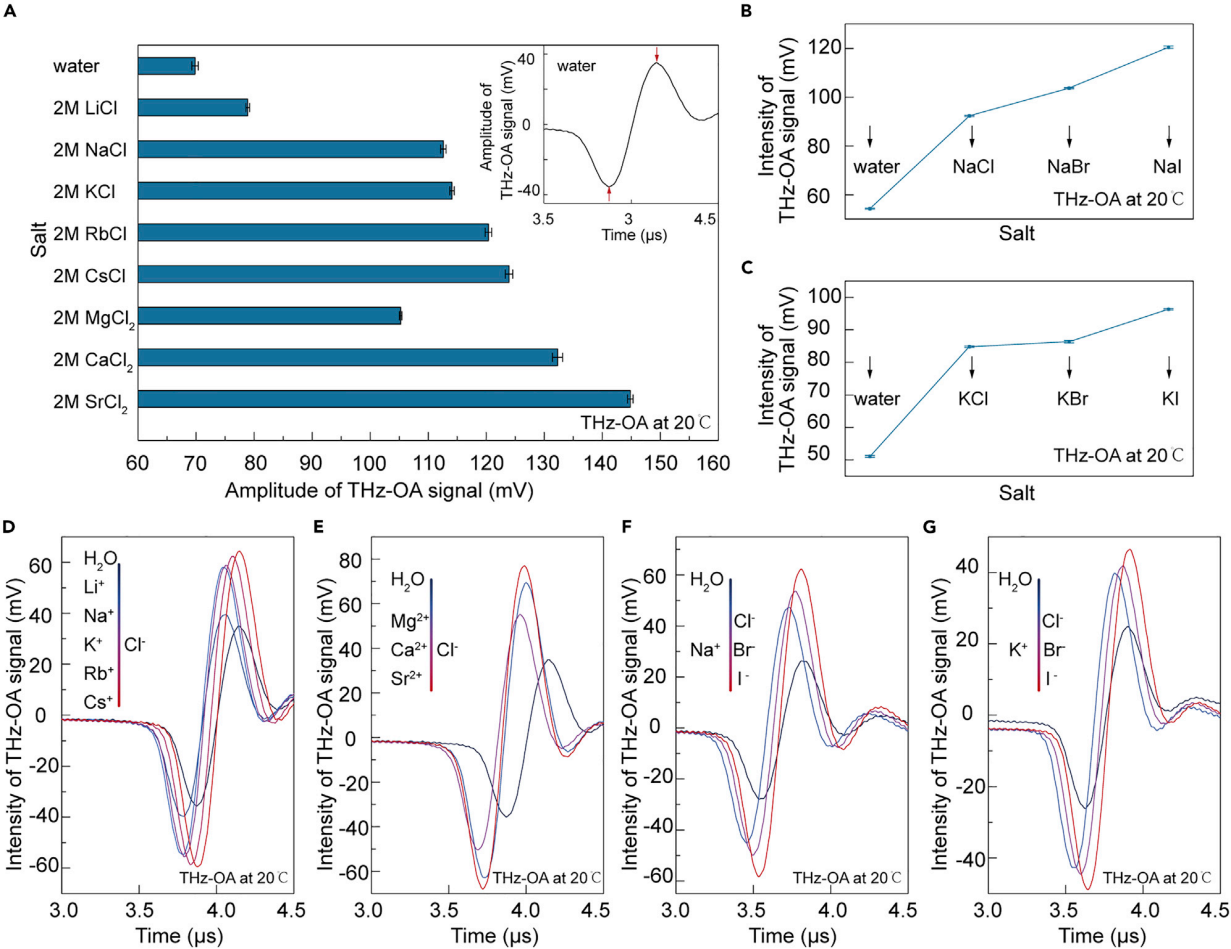
Terahertz optoacoustic (THz-OA) response from water and various salt solutions at 20°C (A) THz-OA signal intensities of water and 2 mol/L aqueous solutions of monovalent and divalent chloride salts at 20°C. Inset: THz-OA response of pure water. (B) THz-OA signal intensities of water and 2 mol/L aqueous solutions of NaCl, NaBr, and NaI at 20°C. (C) THz-OA signal intensities of water and 2 mol/L aqueous solutions of KCl, KBr, and KI at 20°C. (D–G) THz-OA signals of water and different salts solutions in (A)-(C) at 20°C.

First, we detected the time-domain THz-OA signals of pure water and salt solutions of nine alkaline halides and three alkaline earth metal halides, including monovalent and divalent chloride salt solutions, sodium and potassium salt solutions (as shown in Figures 2D–2G). The concentration of these salt solutions was prepared to be 2 mol/L consistently. The monovalent chloride salts included different cations of Li^+^, Na^+^, K^+^, Rb^+^, and Cs^+^. Divalent chloride salts included different cations of Mg^2+^, Ca^2+^, and Sr^2+^. Sodium and potassium salt solutions are with different anions of Cl^−^, Br^−^, I^−^. To quantitatively compare the signal intensity of different salt solutions with that of pure water, the peak-to-peak values of optoacoustic signals were extracted (inset in Figure 2A). Figure 2A shows that the THz-OA signal intensities of monovalent and divalent chloride salt solutions increase in the order of Li^+^ < Na^+^< K^+^< Rb^+^ < Cs^+^ and Mg^2+^ < Ca^2+^ < Sr^2+^, respectively. For sodium and potassium salt solutions, the THz-OA signal intensities show a similar regularity of Cl^−^ < Br^−^ < I^−^ (Figures 2B and 2C). The results demonstrate that the time-domain THz-OA signal intensities of different cations with the same anion or different anions with the same cation in salt solutions vary in consistency with the cation’s or anion’s position in elements groups of the periodic table.

**Figure 3:**
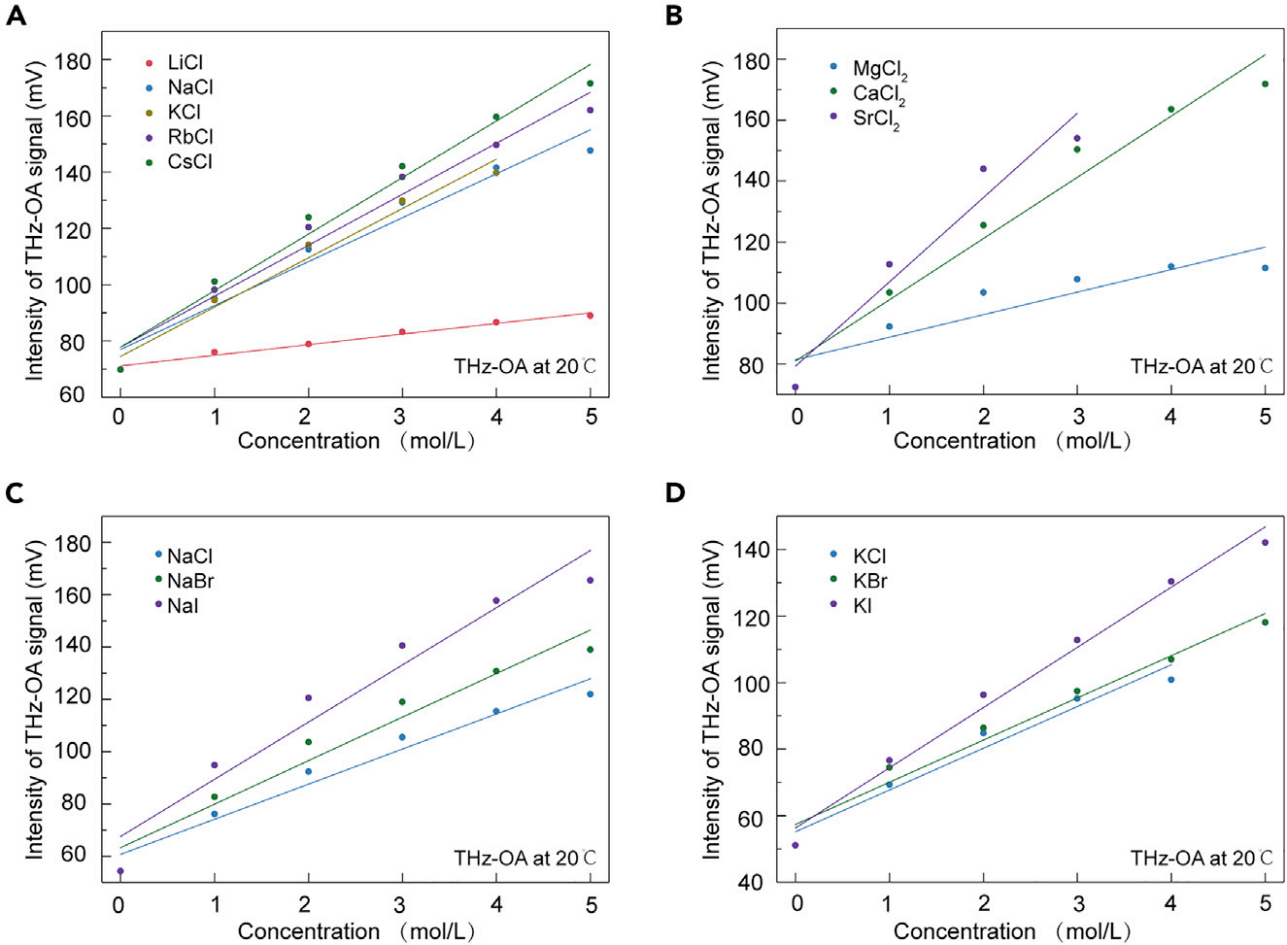
Terahertz optoacoustic (THz-OA) signal intensities of aqueous salt solutions as a function of ion concentrations (A) THz-OA signal intensities of monovalent chloride salt solutions versus ion concentrations. (B) THz-OA signal intensities of divalent chloride salt solutions versus ion concentrations. (C) THz-OA signal intensities of NaCl, NaBr, and NaI solutions versus ion concentrations. (D) THz-OA signal intensities of KCl, KBr, and KI solutions versus ion concentrations. Measurements were taken at 20°C. The solid lines correspond to linear fits.

To investigate the relationship between solute concentrations of salt solutions and time-domain THz-OA signals, we presented the detection of monovalent and divalent chloride salt solutions, sodium and potassium salt solutions with different solute concentrations ranging from 1–5 mol/L at an interval of 1 mol/L. The peak-to-peak values were extracted from THz-OA signals (Figure S1) of different salt solutions and compared versus solute concentrations. It is obvious that the intensities of THz-OA signals increase at a near-linear trend as the increasing of solute concentrations for all kinds of salt solutions (Figure 3). In addition, the slopes of linear fittings are following the order of LiCl < NaCl < KCl < RbCl < CsCl, MgCl_2_ < CaCl_2_ < SrCl_2_, NaCl < NaBr < NaI, and KCl < KBr < KI, respectively.

**Figure 4:**
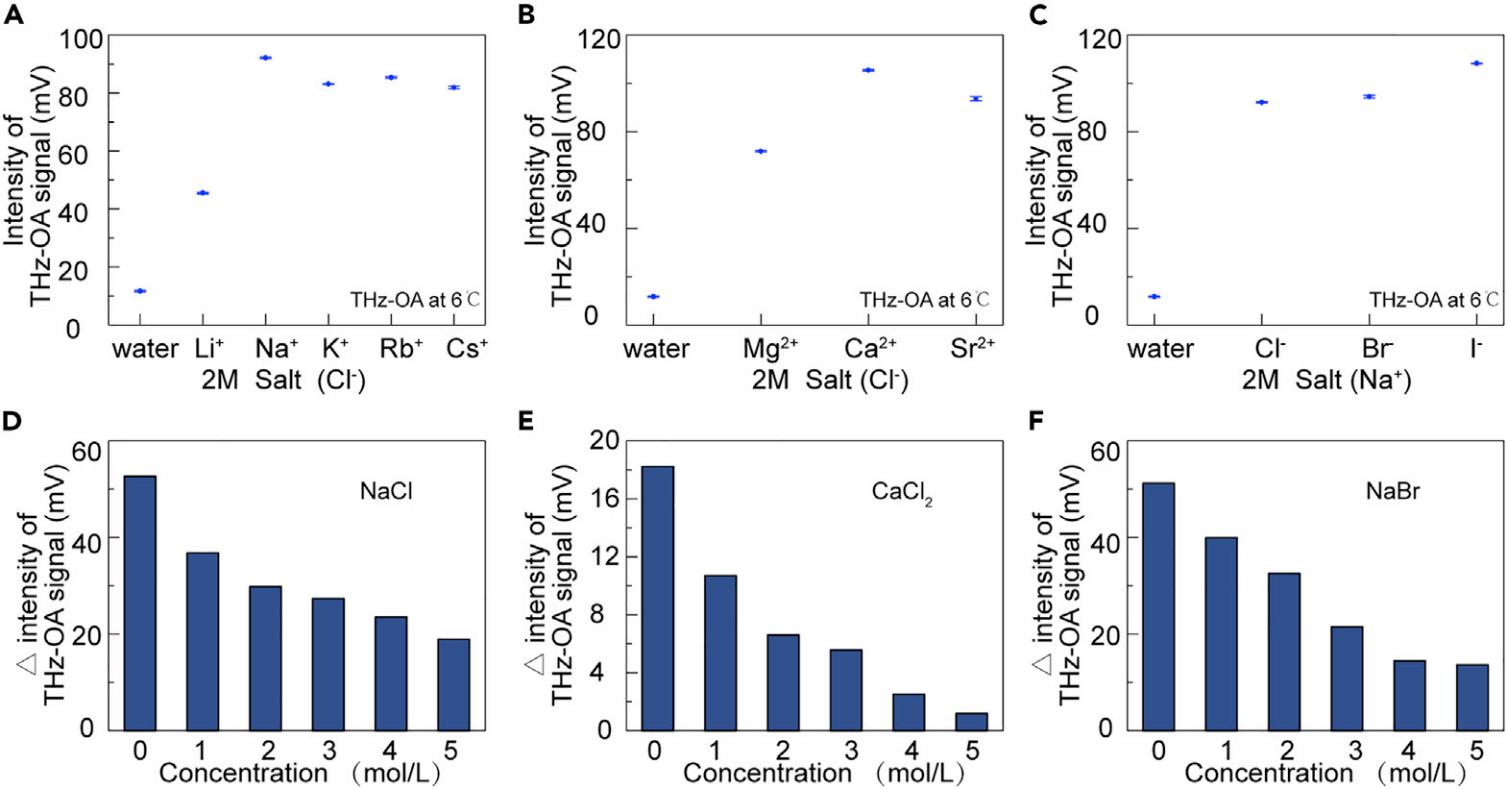
Salt dependence and concentration dependence of terahertz optoacoustic (THz-OA) signals (A) THz-OA signal intensities of water and 2 mol/L aqueous solutions of monovalent chloride salts at 6°C. (B) THz-OA signal intensities of water and 2 mol/L aqueous solutions of divalent chloride salts at 6°C. (C) THz-OA amplitude of water and 2 mol/L aqueous solutions of NaCl, NaBr, NaI at 6°C. (D–F) Concentration dependence of THz-OA signal intensities between 20°C and 6°C of NaCl (D), CaCl_2_ (E), and NaBr (F) solutions with concentrations ranging from 1–5 mol/L.

The previous study has shown that the time-domain THz-OA signals of water could be manipulated through temperature regulation, which can be dampened at low temperature in order to allow sensitive detection of low concentration aqueous salt solutions (Li et al., 2021). To further explore new features of salt solutions with dampened water background, we measured monovalent and divalent chloride salt solutions and sodium salt solutions with the solute concentration of 2 mol/L by THz-OA method at low temperatures. The intensities of THz-OA signals at 6°C are displayed in Figures 4A–4C. Different from the result at room temperature, the THz-OA signals with maximum intensities are from NaCl, CaCl_2_ and NaI solutions, respectively. In addition, LiCl, CaCl_2_, and NaBr solutions at concentrations of 1–5 mol/L were detected to obtain the intensity differences of their THz-OA signals at temperatures between 20°C and 6°C. Figures 4D–4F show that those differences decrease with increasing solute concentration, which may be caused by the different proportions of water in the solutions with different solute concentrations. When the concentration rises, less proportion of water influenced by lowering temperature leads to the smaller intensity differences of THz-OA signals between high and low temperatures.

**Figure 5:**
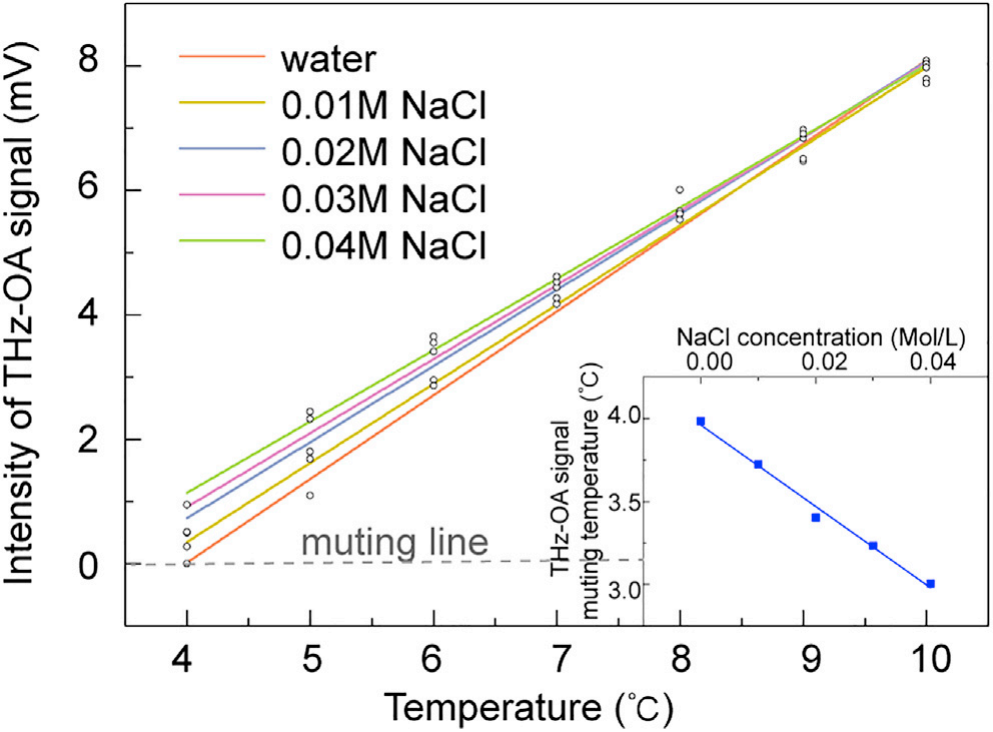
Concentration dependence of the muting temperatures of NaCl solutions Measured terahertz optoacoustic signal intensities of NaCl solutions (with low NaCl concentration of 0.01 (yellow), 0.02 (blue), 0.03 (purple), and 0.04 (green) mol/L) and pure water (red) as a function of temperature. Linear fitting is used on each curve for reading muting temperature (intercept with muting line). Inset: Calculated muting temperatures of NaCl solutions as a function of NaCl concentrations.

To further explore the capability of temperature-controlled time-domain THz-OA detection of salt solutions, we conducted measurements at low temperatures (4–10°C) on NaCl solutions with low concentrations of 0.01, 0.02, 0.03, and 0.04 mol/L, similar to the NaCl concentration in human body (Wishart et al., 2018). Figure 5 shows the extracted peak-to-peak values of THz-OA signals of NaCl solutions and pure water. Linear fitting was used to figure out the muting temperature of each solution (Prakash et al., 2020; Xu et al., 2021). The muting temperatures of aqueous solutions with NaCl concentrations of 0.01, 0.02, 0.03, and 0.04 mol/L were calculated to be 3.73, 3.40, 3.23, and 3.01°C, respectively. A linear fitting with a slope of −23.47°C/(mol/L) and a linear fitting R-square of 0.9807 is acquired (inset in Figure 5).

**Figure 6:**
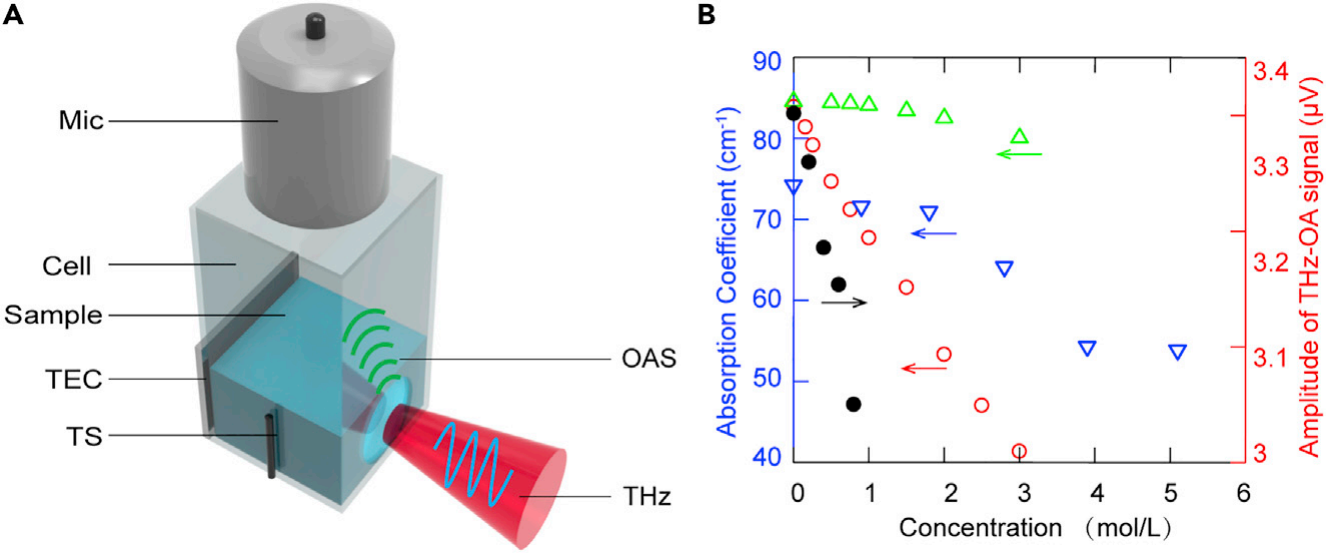
Schematic and experiment result for frequency-domain THz-OA measurement (A) Schematic of the setup. Mic, microphone; OAS, optoacoustic signal; TS, temperature sensor; THz, terahertz radiation; TEC, thermoelectric cooler. (B) THz-OA signal intensities of NaCl solutions at 4°C (black); Absorption coefficient measured in Ref (Vinh et al., 2015) (green), calculated using Double Debye model in Ref (Jepsen and Merbold, 2010) (bule), and calculated using triple Debye model in Ref (Vinh et al., 2015) (red).

Finally, to study the THz absorption of aqueous salt solutions at another THz frequency, we apply frequency-domain THz-OA method to detect pure water and NaCl solutions with relatively low concentration (0.2, 0.4, 0.6, 0.8, mol/L) at 4°C by noncontact measurement. The frequency-domain THz-OA system, as shown in Figure 6A, presented here includes terahertz radiation source, sample cell, temperature control module, and microphone (system details see STAR Methods). The NaCl solution in the cell is illuminated by sinusoidal modulated continuous-wave THz radiation and generates THz-OA signal with the same modulation frequency. Figure 6B shows that the THz-OA signal intensities at 4°C decrease with the increase of NaCl concentration, which corresponds to the trend of absorption coefficient in previous studies (Vinh et al., 2015; Jepsen and Merbold, 2010).

## Discussion and conclusion

We present the THz-OA detection of aqueous solutions of nine alkali halides and three alkaline earth metal halides. Microfluidic chips applied in time-domain THz-OA systems enable dynamic and continuous detection of signals directly induced in water and salt solutions without thickness limitation compared to traditional time-domain THz spectroscopy. Fast and fluent replacement of different salt solutions and consistent background make detection results more credible, and provide potential for real-time or *in vivo* THz-OA detection.

In our study, the THz-OA signals of different monovalent and divalent alkali halide salts vary in consistency with the cation’s and anion’s position in elements groups of the periodic table. The relationship between THz-OA signals and different salt solutions we found provide the potential for identifying salt solutions through simple and convenient THz-OA detection.

To explain the linear increasing trend between time-domain THz-OA signal and solute concentration of salt solutions, we calculate the THz-OA signal of NaCl solutions with different concentrations (0.5, 0.75, 1, 1.5, 2, and 3 mol/L) based on Equation (3). The theoretical calculation result (Figure S2) indicates that in high concentration aqueous solutions, both Grüneisen parameter and absorption coefficient have influence on THz-OA signal, and the linear relationship between THz-OA signal intensity and absorption coefficient in low concentration aqueous solution is broken because of the significant variation of Grüneisen parameter. However, our measurement results of different salt solutions show great regularity and linear relationship with solute concentration, demonstrating a promising label-free tool for distinguishing different salt solutions and quantitative detection of aqueous salt solution with low concentration.

THz-OA signals of aqueous solutions can be manipulated by altering temperature (Li et al., 2021). By lowering the temperature of salt solutions, it is able to mute the THz-OA contribution from water background (Figure 4) and enrich for the contribution from solutes based on Equation (4). Different from traditional THz spectroscopy whose signals of aqueous solutions is mainly contributed by water, the proposed method can extract information of interested solutes through dampening the THz-OA signals of water. This water-manipulated THz-OA method can uniquely achieve sensitivity-enhanced detection of salt solutions with ions' concentration reaching the concentration level in the human body. The THz-OA signals with maximum intensities at low temperatures are from salt solutions containing important ions in life activities, demonstrating the THz-OA method has the great potential to be a powerful tool in biological applications. In addition, the relationship between muting temperatures and salt solutions with low concentrations could be adopted to implement label-free quantitative detection.

Previous studies have shown that the THz absorption of salt solution is concentration and frequency dependent (Jepsen and Merbold, 2010). The THz absorption coefficient of salt solution can be converted by the complex dielectric function through Equation (6), and the complex dielectric function can be described by the Debye model as Equation (5). In Figure 6B, the variation trend of frequency-domain THz-OA signal intensities versus NaCl concentration is consistent with that of absorption coefficient at 0.1 THz in Ref (Vinh et al., 2015) and (Jepsen and Merbold, 2010), which demonstrates that THz-OA method can reflect the THz absorption and can be further used for quantitative spectral analysis in low concentration aqueous solution.

In conclusion, we demonstrate a novel THz-OA method for the dynamic and continuous detection of salt solutions, and investigate the influence of solute concentration, temperature and spectral frequency on THz-OA signal. The proposed method is capable to distinguish salt solutions with different solutes, quantitatively detect and reflect THz absorption of low concentration aqueous solutions. For further study, THz-OA spectroscopy will be developed for deeper understanding of THz absorption of aqueous solution, and THz-OA microscopy will be studied for label-free and real-time imaging of solutes in aqueous solution.

### Limitations of the study

1. In Figure 4, we choose THz-OA signals of aqueous salt solutions at 6°C instead of that at the water-muting temperature which is reported to be 4°C. Although the background signal of water at 6°C is more stable than that around the water-muting temperature, the influence of water absorption still remains for solutions detection.
2. Terahertz radiation source used in the present time-domain THz-OA system has a wide spectrum of 0.2–1.5 THz; therefore, the time-domain THz-OA signals cannot provide the characteristic spectrum of specific ions. By combining the tunable, narrow-spectrum terahertz radiation sources in the future, our time-domain THz-OA method will be able to identify different target molecules based on their THz-OA fingerprints.

## STAR★Methods

### Key resources table

**Table:** 

REAGENT or RESOURCE	SOURCE	IDENTIFIER
**Chemicals, peptides, and recombinant proteins**		

Lithium Chloride	Adamas	CAS#: 7447-41-8
Sodium Chloride	Adamas	CAS#: 7647-14-5
Potassium Chloride	Adamas	CAS#: 7447-40-7
Rubidium Chloride	Adamas	CAS#: 7791-11-9
Cesium Chloride	Adamas	CAS#: 7647-17-8
Magnesium Chloride	Adamas	CAS#: 7786-30-3
Calcium Chloride	Adamas	CAS#: 10043-52-4
Strontium Chloride	Adamas	CAS#: 10476-85-4
Sodium Bromide	Adamas	CAS#: 7647-15-6
Sodium Iodide	Adamas	CAS#: 7681-82-5
Potassium Bromide	Adamas	CAS#: 7758-02-3
Potassium Iodide	Adamas	CAS#: 7681-11-0

**Deposited data**		

Calculation Code	This paper	https://doi.org/10.5281/zenodo.6641966

**Software and algorithms**		

MATLAB (2020b)	MATLAB Software Foundation	https://ww2.mathworks.cn
Origin	Origin Lab	https://www.originlab.com/

### Resource availability

#### Lead contact

Further information and requests for resources and reagents should be directed to and will be fulfilled by the lead contact, Zhen Tian (tianzhen@tju.edu.cn).

#### Materials availability

This study did not generate new unique reagents.

### Experimental model and subject details

Our study does not use experimental models typical in the life sciences.

### Method details

#### System details of time-domain THz-OA

The time-domain THz-OA system is detailed described in our previous work (Li et al., 2021). In order to maximize the THz-OA signal from aqueous solutions and allow the dampening of signal from water background, the THz-OA system presented here incorporates strong-field terahertz radiation source, piezoelectric ultrasonic transducer, customer-build sample holder and temperature control module (Figure 1A). The terahertz pulses were generated using the tilted-pulse-front technique. A femtosecond Ti: sapphire regenerative amplifier (Coherent, USA) pumped the nonlinear crystal LiNbO_3_ with a pulse front tilt of 63° (Figure 1A). The duration time and repetition rate of the pump laser beam are 35 fs and 1 kHz at a wavelength of 800 nm, respectively. The generated terahertz beams with the energy per pulse of 4 μJ and spectral range 0.2–1.5 THz are then collimated and focused to a spot of ∼1.5 mm by off-axis parabolic mirrors. The radiant exposure is 0.23 mJ/cm^2^, which satisfies the confinement conditions for optoacoustics and fall within the laser exposure limit of 20 mJ/cm^2^. In order to detect samples in liquid state conveniently, customized microfluidic chips were made with a center circle detection area (∼3 mm diameter) and channels on both sides (∼2 mm width). The chip has a rear surface of PDMS (5 mm thick) and a front surface of quartz (300 μm), which was pasted on the sample holder with centers aligned (Figure 1A). Upon absorption of terahertz radiation, aqueous media heat up, inducing thermoelastic expansion that produces acoustic waves that can be detected, which is referred to as the THz-OA effect. THz-OA signals were detected using flat piezoelectric ultrasonic transducers (Olympus, USA, 2.25 MHz central frequency) coupled with the chip’s rear surface of by ultrasound gel, amplified by a low-noise 50-dB amplifier (Usultratek, USA), digitized at a sampling rate of 200 MS/s using a data acquisition card (Gage, USA). The temperature of the sample within the chip was controlled using a custom-built temperature control module (Figure 1B) (Li et al., 2021). It can provide temperature regulation ranging from −10°C to 50°C approximately.

#### System details of frequency-domain THz-OA

The frequency-domain THz-OA system is shown in Figure 6A. The continuous-wave terahertz radiation is generated by a sub-terahertz source (TeraSense, USA) and modulated at 5 kHz by an arbitrary function generator (Tektronix, USA). The output frequency of this source is 0.1 THz, and the average power is 80 mW. The THz-OA signal was detected using a microphone (BSWA, China) and processed by a lock in amplifier (Zurich Instruments, Switzerland). The temperature of the sample within the cell was controlled by a temperature control module, which is mentioned above.

#### Sample preparation

All salts we used have the highest available purity and are listed in the key resources table. In order to prepare salt solutions with specific concentrations, the mass of solute is calculated according to the relative molecular mass. Then, the solute is weighed with a high-precision balance and completely dissolved with deionized water in a volumetric flask.

#### Principle of THz-OA

The generation and propagation of optoacoustic signal induced by a short electromagnetic pulse can be described by optoacoustic equation as (Wang and Wu, 2008)

(Equation 1) $$\left(\nabla^{2}-\frac{1}{V_{S}^{2}}\frac{\partial^{2}}{\partial t^{2}}\right)P\left(\vec{r},\;t\right)=-\frac{\beta }{C_{p}}\frac{\partial H\left(\vec{r},\;t\right)}{\partial t}$$

where *V*_*S*_ is the speed of sound, *C*_*P*_ is the specific heat capacity, *β* is the thermal coefficient of volume expansion, and *H* is the heating source. When the pulse width is much less than the thermal relaxation time and pressure relaxation time, the local pressure rise after the laser excitation pulse can be written as

(Equation 2) $$P_{0}=\Gamma \eta_{th}\mu_{a}F=\left(\frac{\beta V_{S}^{2}}{C_{P}}\right)\eta_{th}\mu_{a}F$$

where *Γ* is the dimension-less Grüneisen parameter. The factor *μ*_*a*_ represents the optical absorption coefficient, which is determined by the absorption characteristics of the material at the given frequency of electromagnetic wave, *F* shows the optical fluence (the optical energy per unit area) and *η*_*th*_ defines the percentage of absorbed energy converted into heat. Since the terahertz pulse we use in time-domain THz-OA has a spectral range, the local pressure should be exactly written as

(Equation 3) $$P_{0}=\left(\frac{\beta V_{S}^{2}}{C_{P}}\right)\eta_{th}\int_{0.2THz}^{1.5THz}\mu_{a}\left(v\right)F_{v}\left(v\right)dv$$

where *Fv*(*v*), *μ*_*a*_(*v*) is the distribution of optical fluence and absorption coefficient at a given spectral frequency *v*, respectively.

#### Principle of water muting

At 4 ^o^C, due to *β*_water_ = 0 for water, the local pressure of aqueous solution based on water muting method and Despretz law can be further written as (Prakash et al., 2020)

(Equation 4) $$P_{0}=\beta_{2}Kc\frac{V_{S}^{2}}{C_{p}}\eta_{th}\int_{0.2THz}^{1.5THz}\mu_{a}\left(v\right)F_{v}\left(v\right)dv$$

where *c* is the solute concentration, and *β*_2_, *K* is a constant in thermal coefficient of volume expansion and Despretz law respectively, which are determined by aqueous solution. In different low concentration aqueous solution, the change of *β*_2_, *K*, *V*_*S*_, *C*_*P*_, *η*_*th*_ and *μ*_*a*_ can be ignored (Wang and Wu, 2008). The local pressure at water muting temperature is proportional to the solute concentration and can be used for quantitative detection analysis.

The optoacoustic pressure of samples illuminated by terahertz pulses leads to the generation of optoacoustic signals. The THz-OA signal relates to the Grüneisen parameter, absorbing materials and characteristics of terahertz radiation (Ntziachristos, 2010). In aqueous solutions, these parameters are functions of both temperature and solute concentration (Darros-Barbosa et al., 2003). In this article, we use THz-OA method to measure salt solutions with different cation-anion pairs, different ion concentrations and temperatures.

#### Debye model

The complex dielectric function, $\varepsilon \left(v\right)=\varepsilon^{'}\left(v\right)+i\varepsilon^{''}\left(v\right)$, of polar liquids can be described by Debye model as (Vinh et al., 2015)

(Equation 5) $$\varepsilon \left(v\right)=\varepsilon_{\infty }+\sum_{j=1}^{N}\frac{\Delta \varepsilon_{j}}{1-2i\pi v\tau_{j}}+\frac{i\sigma_{s}}{2\pi \varepsilon_{0}}$$

where *ε*_∞_ is the high-frequency contribution to the complex dielectric function, Δ*ε*_*j*_, *τ*_j_ is the dielectric strength and relaxation time of the *j*th relaxation process respectively, *σ*_*s*_ is the static conductivity of the electrolyte solution. The relation between complex dielectric function and absorption coefficient is given as (Vij et al., 2004)

(Equation 6) $$\mu_{a}\left(v\right)=\frac{4\pi v}{C_{0}}{\left(\frac{\sqrt{\varepsilon^{'}{\left(v\right)}^{2}+\varepsilon^{''}{\left(v\right)}^{2}}}{2}-\frac{\varepsilon^{'}\left(v\right)}{2}\right)}^{1/2}$$

where *C*_0_ is the speed of light in vacuum. The solute concentration has influence on dielectric properties and THz absorption of aqueous solution (Jepsen and Merbold, 2010). In order to figure out the effect of solute concentration on THz absorption, we measure NaCl solutions with different concentrations at water muting temperature via frequency-domain THz-OA method.

### Quantification and statistical analysis

There is no statistical analysis or quantification in this paper.

### Additional resources

We have no relevant resources.

## Data Availability

• All data reported in this paper will be shared by the lead contact upon request. • All original code has been deposited at Zenodo and is publicly available as of the date of publication. DOI is listed in key resources table. • Any additional information required to reanalyze the data reported in this paper is available from the lead contact upon request.
