# Supplementary material for: Terahertz optoacoustic detection of aqueous salt solutions
Source: iScience. 2022 Jun 26;25(7):104668. doi: 10.1016/j.isci.2022.104668 (PMC9272373; doi:10.1016/j.isci.2022.104668)
Supplement: Document S1. Figures S1 and S2 [file mmc1.pdf]

iScience, Volume *25*

Supplemental information

Terahertz optoacoustic detection of aqueous salt solutions

Liwen Jiang, Ke Zhang, Yixin Yao, Shuai Li, Jiao Li, Zhen Tian, and Weili Zhang

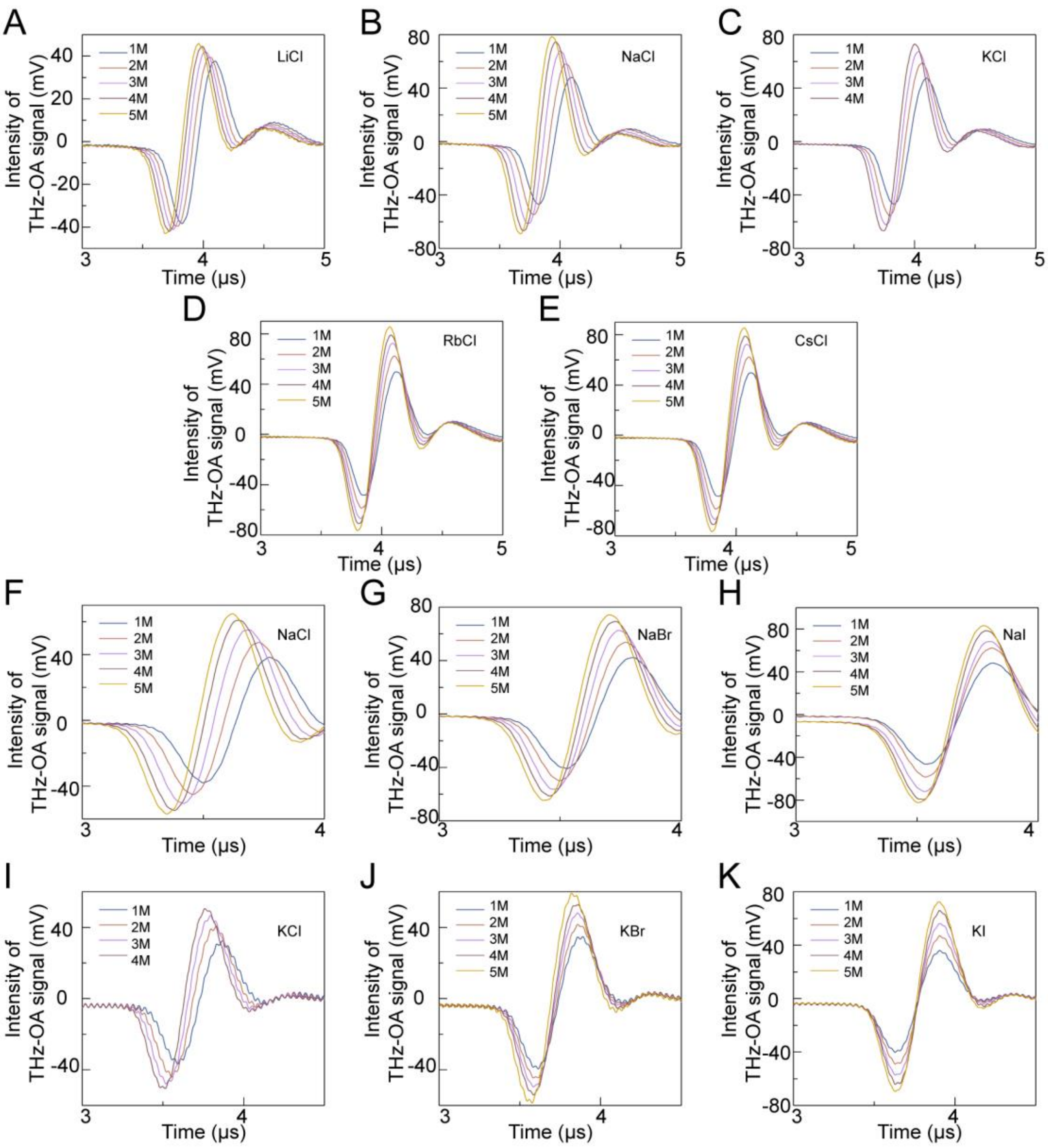

Figure S1. Concentration dependence of the time-domain THz-OA signal of different salt solutions at 20 °C, Related to Figure 3. THz-OA response of increasingly concentrated LiCl (A), NaCl (B) (F), KCl (C) (I), RbCl (D), CsCl (E), NaBr (G), NaI (H), KBr (J) and KI (K) solutions.

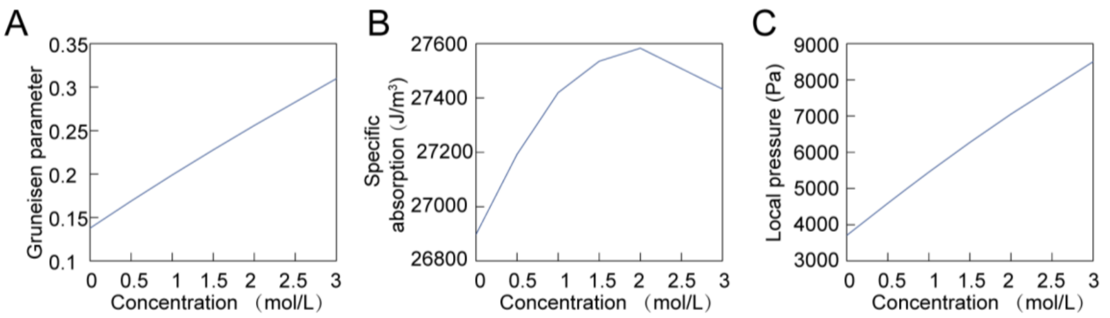

Figure S2. Theoretical calculation result of time-domain THz-OA signal intensities of NaCl solutions with different concentration, Related to Figure 3. (A) Grüneisen parameter versus NaCl concentrations. (B) Specific absorption versus NaCl concentrations. (C) Local pressure versus NaCl concentrations. The absorption coefficient data of NaCl solutions we use is measured in Ref (Vinh, Sherwin, Allen, George, Rahmani and Plaxco, 2015).
